# Elimination of senescent osteoclast progenitors has no effect on the age‐associated loss of bone mass in mice

**DOI:** 10.1111/acel.12923

**Published:** 2019-02-17

**Authors:** Ha‐Neui Kim, Jianhui Chang, Srividhya Iyer, Li Han, Judith Campisi, Stavros C. Manolagas, Daohong Zhou, Maria Almeida

**Affiliations:** ^1^ Division of Endocrinology and Metabolism, Center for Osteoporosis and Metabolic Bone Diseases University of Arkansas for Medical Sciences Little Rock Arkansas; ^2^ The Central Arkansas Veterans Healthcare System Little Rock Arkansas; ^3^ Department of Pharmaceutical Sciences and Winthrop P. Rockefeller Cancer Institute University of Arkansas for Medical Sciences Little Rock Arkansas; ^4^ Department of Orthopedic Surgery University of Arkansas for Medical Sciences Little Rock Arkansas; ^5^ Buck Institute for Research on Aging Novato California; ^6^ Lawrence Berkeley National Laboratory Berkeley California; ^7^ Department of Pharmacodynamics, College of Pharmacy University of Florida Gainesville Florida

**Keywords:** aging, osteoblasts, osteocytes, osteoporosis, p16

## Abstract

Both an increase in osteoclast and a decrease in osteoblast numbers contribute to skeletal aging. Markers of cellular senescence, including expression of the cyclin inhibitor p16, increase with aging in several bone cell populations. The elimination of p16‐expressing cells in old mice, using the INK‐ATTAC transgene, increases bone mass indicating that senescent cells contribute to skeletal aging. However, the identity of the senescent cells and the extent to which ablation of p16‐expressing cells may prevent skeletal aging remain unknown. Using mice expressing the p16‐3MR transgene, we examined whether elimination of p16‐expressing cells between 12 and 24 months of age could preserve bone mass; and whether elimination of these cells from 20 to 26 months of age could restore bone mass. The activation of the p16‐3MR transgene by ganciclovir (GCV) greatly diminished p16 levels in the brain, liver, and osteoclast progenitors from the bone marrow. The age‐related increase in osteoclastogenic potential of myeloid cells was also abrogated by GCV. However, GCV did not alter p16 levels in osteocytes—the most abundant cell type in bone—and had no effect on the skeletal aging of p16‐3MR mice. These findings indicate that the p16‐3MR transgene does not eliminate senescent osteocytes but it does eliminate senescent osteoclast progenitors and senescent cells in other tissues, as described previously. Elimination of senescent osteoclast progenitors, in and of itself, has no effect on the age‐related loss of bone mass. Hence, other senescent cell types, such as osteocytes, must be the seminal culprits.

## 1 INTRODUCTION

Soon after the attainment of peak bone mass, the balance between bone resorption and bone formation begins to progressively tilt in favor of the former, in both women and men (Bala, Zebaze, & Seeman, 2015; Looker et al., 1993; Riggs et al., 2008). Both female and male mice exhibit all seminal features of skeletal aging found in humans, including the decline of trabecular and cortical bone mass (Almeida et al., 2007; Glatt, Canalis, Stadmeyer, & Bouxsein, 2007; Piemontese et al., 2017). The age‐related cortical bone loss in mice is associated with an increase in the number of osteoclasts, the cells responsible for degrading the bone matrix (Piemontese et al., 2017; Ucer et al., 2017). Osteoclasts differentiate from myeloid lineage cells in response to osteoclastogenic signals such as M‐CSF and RANKL. Nonetheless, a decline in bone formation is the seminal culprit of skeletal aging in both humans and rodents (Almeida et al., 2007; Parfitt, Villanueva, Foldes, & Rao, 1995). A decrease in the number of osteoblasts, the cells that synthesize the bone matrix, underlies the loss of both trabecular and cortical bone in aged mice (Almeida et al., 2007). Osteoblasts differentiate from mesenchymal progenitors, and this process is dependent on the activity of the transcription factors Runx2 and Osx1 (Park et al., 2012). Importantly, the number of these osteoprogenitors declines with advancing age and this decline is associated with increased markers of cellular senescence (Kim et al., 2017).

Cellular senescence is characterized by a permanent proliferative arrest, and an altered gene expression pattern leading to the production of pro‐inflammatory and matrix‐degrading molecules known as the senescence‐associated secretory phenotype (SASP) (Campisi, 2013; Coppe et al., 2008; Kuilman et al., 2008). Osteocytes, former osteoblasts buried in the bone matrix, are postmitotic and the most abundant cell type in bone (Jilka & O'Brien, 2016). Osteocytes modulate bone resorption and formation via the production of RANKL and Sost, respectively (Baron & Kneissel, 2013; O'Brien, Nakashima, & Takayanagi, 2013). Earlier findings by us and others have elucidated that, like other postmitotic cells, osteocytes in the bone of aged female and male mice show markers of senescence (Farr et al., 2016; Piemontese et al., 2017).

Several, but not all, senescent cell types exhibit high levels of the cyclin inhibitor p16. For example, senescent osteocytes have increased levels of p16, while senescent osteoblast progenitors have elevated levels of p21, but not p16 (Kim et al., 2017; Piemontese et al., 2017). Selective elimination of cells expressing p16 in mouse models increases life‐ and healthspan (Childs et al., 2017). Currently, two of such models have been described: the INK‐ATTAC and the p16‐3MR mice (Baker et al., 2011; Demaria et al., 2014). Using the p16‐3MR model, we have effectively depleted senescent cells in the skin, lungs, muscle, and bone marrow, including senescent hematopoietic and muscle stem cells, and suppressed the SASP in either sub‐lethally irradiated or normally aged mice (Chang et al., 2016; Demaria et al., 2014). Farr and colleagues have found that elimination of p16‐expressing cells in 20‐month‐old mice for a 4‐month period, using the INK‐ATTAC transgene, increases bone mass (Farr et al., 2017). These findings support the notion that senescent cells contribute to age‐related bone loss. However, the identity of the senescent cells that are responsible for skeletal aging remains unknown. Likewise, the extent to which elimination of p16‐expressing cells rescues skeletal aging is unknown. Here, we investigated the skeletal effects of long‐ term ablation of senescent cells using p16‐3MR mice—an alternative to the INK‐ATTAC model of p16‐expressing cell elimination in which the transgene is activated by the administration of GCV. The key objectives of this work were two: first, to eliminate p16 senescent cells from 12 to 24 months of age, the time period during which C57BL/6 mice experience a dramatic age‐related loss of bone mass (Almeida et al., 2007), and determine whether the experimental maneuver could prevent the loss of bone. And second, to eliminate p16 senescent cells from 20 to 26 months of age in order to determine whether this intervention could restore bone mass in mice that had already lost it.

## 2 RESULTS

### 2.1 GCV administration to p16‐3MR mice eliminates senescent cells from the brain and liver, but not osteocytes

**Figure 1:**
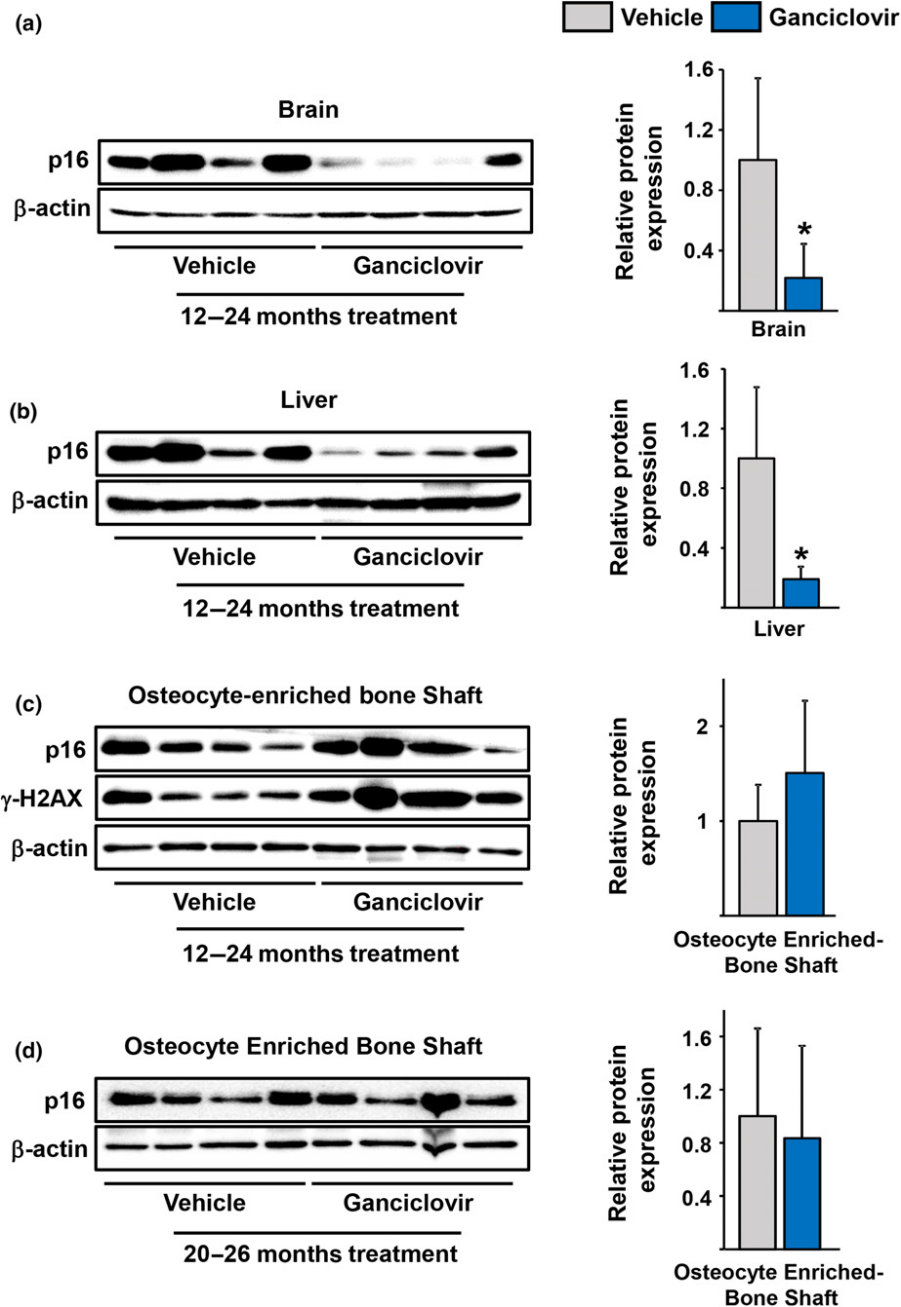
Levels of p16 in several tissues of old p16‐3MR mice. (a–d) Protein was isolated from the indicated tissues of (a–c) 24‐month‐old mice which received vehicle or Ganciclovir for 12 months, and (d) 26‐month‐old mice which received vehicle or Ganciclovir for 6 months. Each lane on the immunoblots represents one mouse (brain and liver, *n* = 4; bone shaft, *n* = 9). On the right, the expression of p16 was calculated as a ratio to β‐actin levels within in each lane. *p < 0.05 by Student's *t* test. Bars represent mean and *SD* (*error bars*)

We administered GCV or PBS (vehicle) to p16‐3MR female mice, in the C57BL/6 genetic background, from 12 to 24 months of age (Supporting information Figure S1b). Mice receiving GCV exhibited the same weight at 24 months as the control mice (data not shown). As expected, p16 protein levels in the brain and liver of mice treated for one year with GCV were greatly diminished (Figure 1a and b). However, p16 protein levels were not affected in osteocyte‐enriched bone shafts (Figure 1c), indicating that the 3MR transgene does not ablate p16‐expressing osteocytes. We have previously shown that the increase in p16 levels in cortical bone osteocytes is associated with increased levels of γH2AX, a marker of DNA damage (Piemontese et al., 2017). Consistent with the lack of an effect on p16 levels, GCV administration did not alter γH2AX in osteocytes (Figure 1c).

In a second study, we administered GCV or PBS to p16–3MR female mice from 20 to 26 months of age (Supporting information Figure S1c). At 20 months of age, mice have already experienced significant loss of bone mass (Almeida et al., 2007; Piemontese et al., 2017). Like the findings in our first study, the levels of p16 in the cortical bone of GCV‐treated mice were undistinguishable from the vehicle‐treated mice at 26 months (Figure 1d).

### 2.2 GCV administration to p16‐3MR mice eliminates senescent osteoclast progenitors

**Figure 2:**
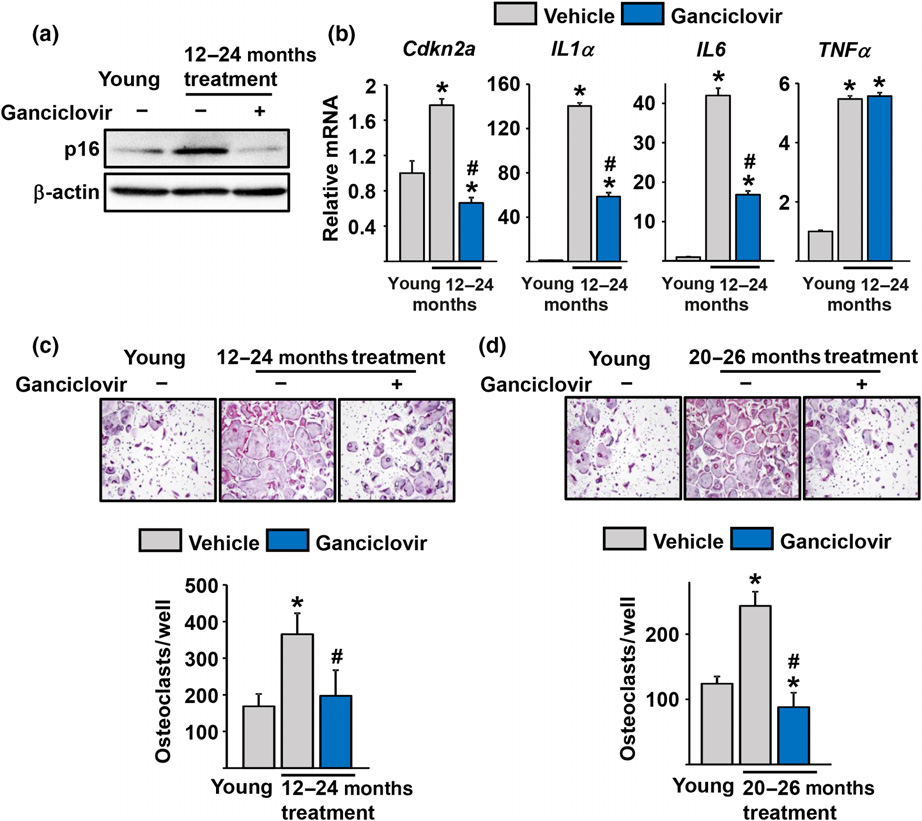
Activation of the p16‐3MR transgene eliminates osteoclast progenitors expressing p16. (a–c) Cultures of BMMs isolated from 24‐month‐old female p16‐3MR mice administered vehicle or Ganciclovir (*n* = 9 mice/group) for 12 months. (d) Cultures of BMMs isolated from 26‐month‐old female p16‐3MR mice were either vehicle‐ or Ganciclovir‐administered (*n* = 7 mice/group) for 6 months. Three‐month‐old female p16–3MR mice were used as a control. (a) p16 levels detected by Western blot and (b) p16 and SASP levels detected by qRT‐PCR after 48 hr in the presence of M‐CSF and (c and d) *Top*, Representative pictures and *Bottom*, number of TRAP‐positive multinucleated osteoclasts derived from BMMs of the mice cultured with M‐CSF (30 ng/ml) and RANKL (30 ng/ml) for 5 days (triplicates). *p < 0.05 vs. young mice, ^#^ *p* < 0.05 vs. vehicle‐treated old mice; unpaired Student's *t* test. Bars represent mean and S.D. (*error bars*)

Because the osteoclastogenic potential of myeloid cells and the number of osteoclast in endocortical bone increases with age, we next examined the levels of p16 in osteoclast progenitors. To do this, we obtained bone marrow‐derived macrophages (BMMs) from mice in which the p16‐3MR transgene was activated from 12 to 24 month of age and cultured them in the presence of M‐CSF (Figure 2a). Cultures from 3‐month‐old mice were used as young controls. P16 protein levels were greatly increased in 24‐month‐old p16‐3MR mice receiving vehicle. In contrast, cells from 24‐month‐old p16‐3MR mice receiving GCV had similar p16 levels to those of young mice (Figure 2a). The mRNA levels of Cdkn2a (the gene that encodes both p16Ink4a and p19Arf) and common elements of the SASP such as IL‐1α, IL‐6, and TNFα were also increased with age in the osteoclast progenitor cultures; and all, except TNFα, were greatly decreased by GCV administration (Figure 2b). Likewise, the number of osteoclasts formed in cultures of BMMs from the aged control mice was much greater than that the young, and this increase was also prevented by elimination of senescent cells with GCV (Figure 2c). Administration of GCV to wild‐type mice had no impact on osteoclast formation in vitro (data not shown).

The administration of GCV to 20‐month‐old p16‐3MR mice for 6 months also caused a decline in the number of osteoclast formed from BMM cultures obtained when mice were sacrificed at 26 months (Figure 2d).

### 2.3 GCV administration to p16‐3MR mice decreases the adipogenic potential but does not alter the osteoblastogenic potential of bone marrow stromal cells

**Figure 3:**
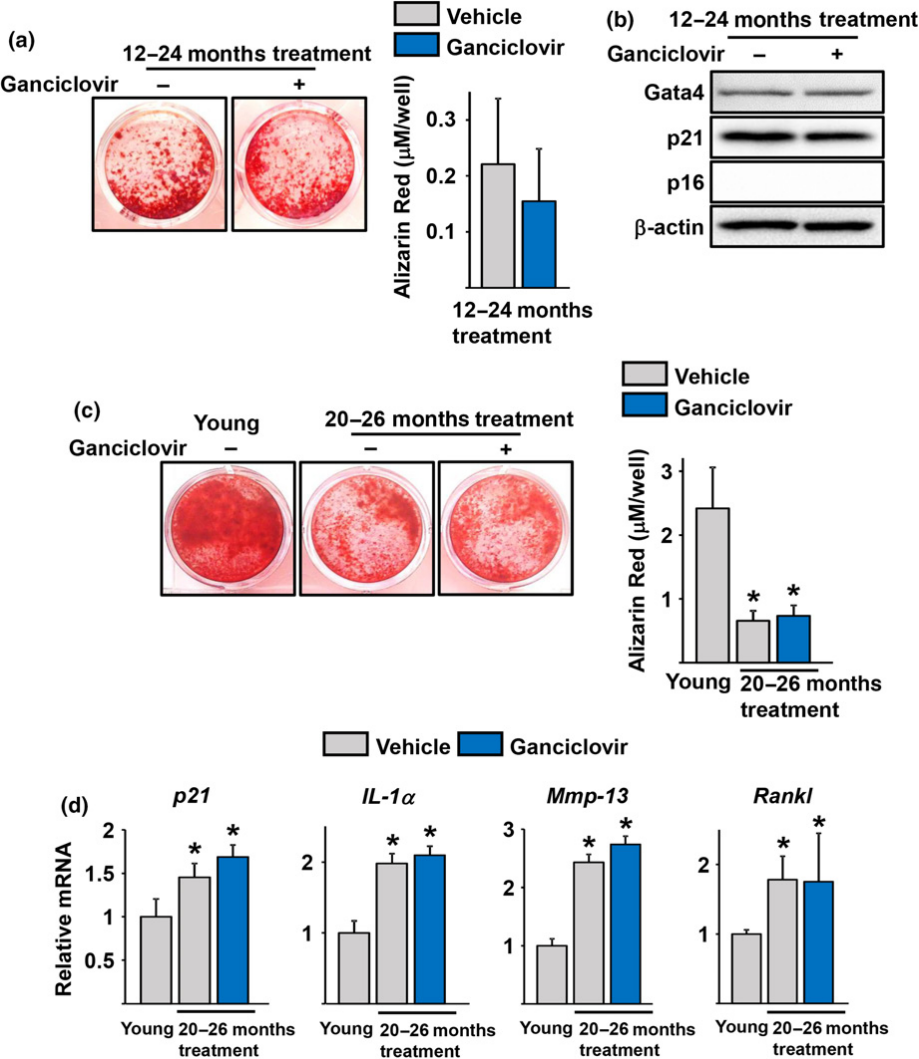
Activation of the p16‐3MR transgene does not alter osteoblastogenesis. (a‐d) Bone marrow stromal cells were isolated from 24‐ (a and b) and 26‐month‐old (c and d) female p16‐3MR mice received vehicle or Ganciclovir (*n* = 7–9 mice/group) for 12 months (a and b) or 6 months (c and d) and cultured with ascorbate and β‐glycerophosphate (triplicates). (a and c) *Left*, Representative pictures and *Right*, quantification of Alizarin Red Staining in cells cultured for 21 days and (b) protein levels by Western blot and (d) mRNA levels by qRT‐PCR in cells cultured for 7 days. *p < 0.05 vs. young mice; unpaired Student's *t* test. Bars represent mean and *SD* (*error bars*)

We next examined whether ablation of p16‐expressing cells in the bone marrow of p16‐3MR mice altered osteoblast or adipocyte formation in culture. Bone marrow stromal cells from 24‐month‐old p16‐3MR mice treated with GCV showed the same degree of mineralization as cells from mice treated with vehicle when cultured under osteogenic conditions (Figure 3a). As described before (Kim et al., 2017), p16 protein was undetectable in stromal cells from old mice (Figure 3b). Nevertheless, the levels of p21, GATA4, and SASP are elevated in osteoblast progenitors from old mice (Kim et al., 2017). GCV administration to p16‐3MR mice did not alter the levels of p21 or GATA4 (Figure 3b). Mineralization was also unaffected in bone marrow cultures from 26‐month‐old mice in which the p16‐3MR was activated for 6 months (Figure 3c). Cultures from 3‐month‐old mice were used as young control. Similar to earlier findings (Kim et al., 2017), the mRNA levels of p21, IL‐1α, Mmp‐13, and RANKL were increased with age in the stromal cell cultures (Figure 3d). GCV administration to mice did not alter the expression of any of these genes (Figure 3d).

**Figure 4:**
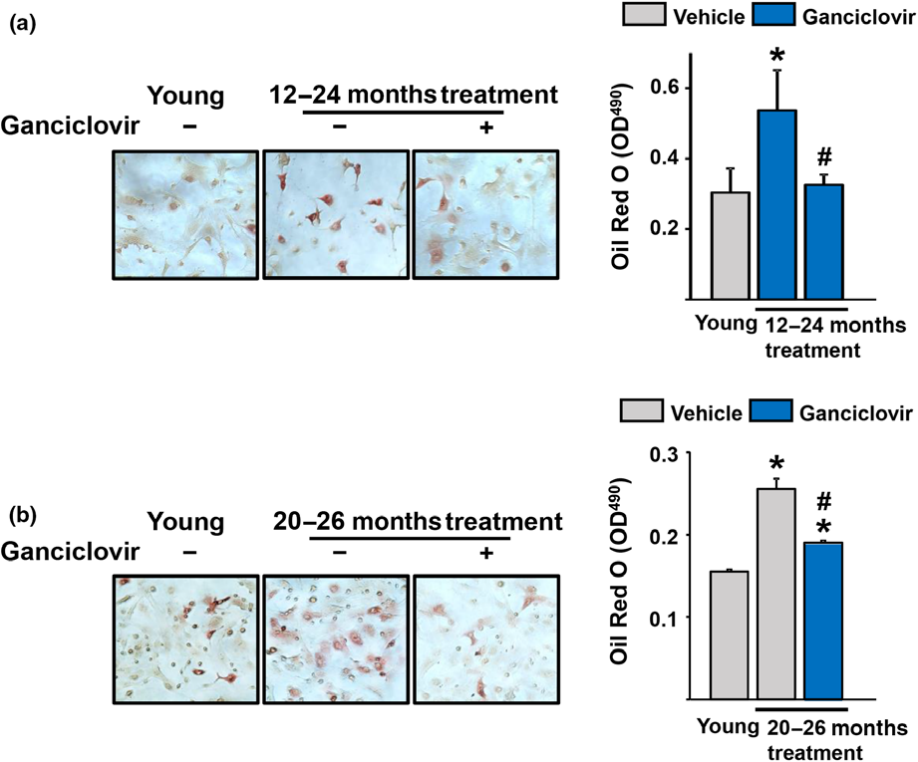
Activation of the p16‐3MR transgene prevents the increase in adipocyte precursors in the bone marrow. (a and b) Bone marrow stromal cells were isolated from 24‐ (a) and 26‐month‐old (b) female p16‐3MR mice received vehicle or Ganciclovir (*n* = 7–9 mice/group) for 12 months (a) or 6 months (b) and cultured with rosiglitazone for 14 days (triplicates). *Left*, Representative pictures and *Right*, quantification of Oil Red O Staining in cells. *p < 0.05 vs. young mice, ^#^ *p* < 0.05 vs. vehicle‐treated old mice; unpaired Student's *t* test. Bars represent mean and *SD* (*error bars*)

Marrow adipocytes increase with aging in humans and rodents (Horowitz et al., 2017). Accordingly, the number of adipocytes formed in bone marrow cultures is higher in cells obtained from old than young mice (Moerman, Teng, Lipschitz, & Lecka‐Czernik, 2004). GCV administration decreased the adipogenic potential of bone marrow cells (Figure 4a–b). Specifically, the number of Oil Red O‐positive cells, formed in cultures of stromal cells in the presence of the PPARγ stimulator rosiglitazone, was higher in cells obtained from p16‐3MR mice receiving vehicle when compared to cultures from young mice. The number of adipocytes was reduced in cultures derived from mice treated for 12 (Figure 4a) or 6 months (Figure 4b) with GCV, when compared to cells obtained from the age‐matched control mice.

### 2.4 Elimination of senescent cells with p16‐3MR does not affect the age‐associated loss of bone mass or strength

**Figure 5:**
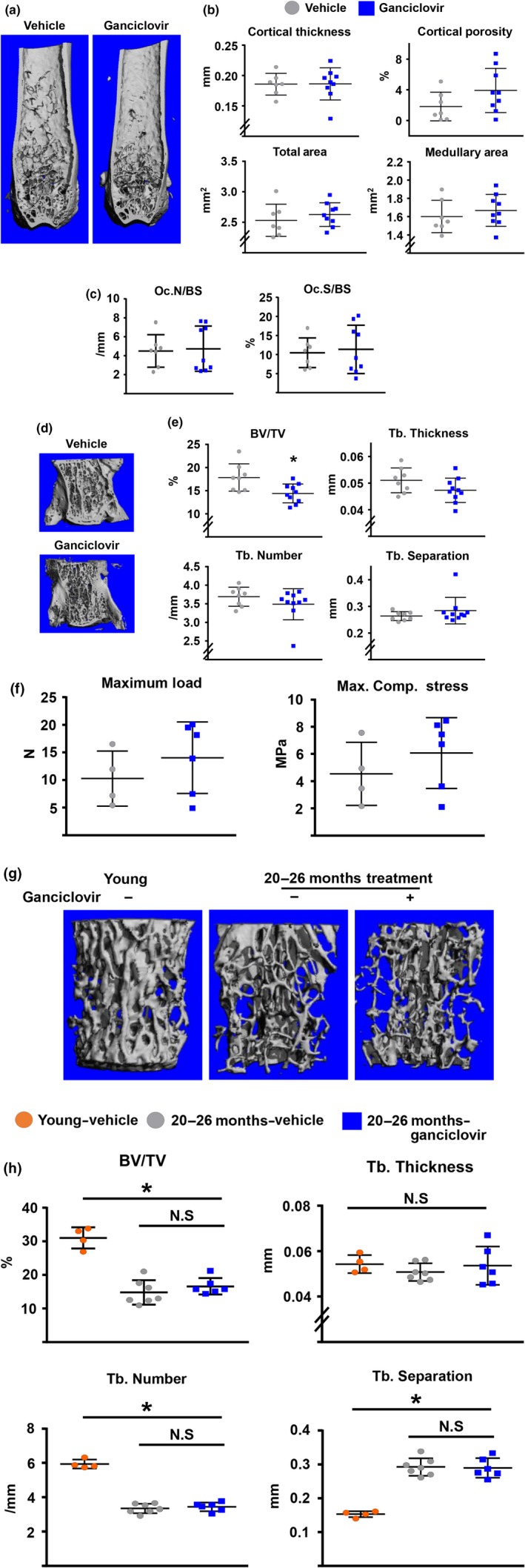
Activation of the p16‐3MR transgene does not affect bone mass in aged mice. (a–f) 12‐month‐old female p16‐3MR mice were either vehicle‐administered or Ganciclovir‐administered (*n* = 9) for 12 months. (a) Representative micro‐CT images of femur. (b) Femoral cortical thickness, total area, and medullary area by micro‐CT in the midshaft region and cortical porosity in the distal metaphysis. (c) Osteoclast number (Oc. N) and surface (Oc. S) at the endocortical area of the femurs. (d) Representative micro‐CT images of L5 vertebrae. (e) Vertebral trabecular bone parameters (Bone volume per tissue volume, BV/TV; Trabecular Thickness, Tb. Thickness; Trabecular Number, Tb. Number; Trabecular Separation, Tb. Separation) as determined by micro‐CT. (f) Load‐to‐failure, a measure of strength, was determined by compression testing of L1 vertebrae. Maximum load (*left*) and Maximum compressive stress (*Right*). (g and h) 20‐month‐old female p16–3MR mice were either vehicle‐administered (*n* = 7) or Ganciclovir‐administered (*n* = 7) for 6 months. (g) Representative micro‐CT images of L5 vertebrae. (h) Vertebral trabecular bone parameters (Bone volume per tissue volume, BV/TV; Trabecular Thickness, Tb. Thickness; Trabecular Number, Tb. Number; Trabecular Separation, Tb. Separation) as determined by micro‐CT. Three‐month‐old female p16‐3MR mice (*n* = 4) were used as a young control group. *p < 0.05 vs. young mice; unpaired Student's *t* test. Data represent mean and *SD* (*error bars*)

To examine the skeletal effects of the p16‐3MR transgene, we used micro‐CT. Cortical bone mass, measured in the midshaft of the femur, was not different in 24‐month‐old mice receiving GCV for 12 months, when compared to mice receiving vehicle (Figure 5a,b). In line with the lack of an effect on bone mass, the number of osteoclasts at the endocortical surface of the femurs was not altered by GCV (Figure 5c). Similarly, trabecular bone mass and microarchitecture, measured in the vertebrae, were unaffected by the activation of the transgene with GCV (Figure 5d and e). Because bone strength can be altered independently of bone mass, we measured bone strength using compression testing. Maximum load and maximum compression stress in the vertebrae were indistinguishable between vehicle‐ and GCV‐treated p16‐3MR mice (Figure 5f). To determine whether GCV could exert effects independently of the activation of the p16‐3MR transgene, we administered GCV to wild‐type C57BL/6 for the same period of time. The administration of GCV to wild‐type mice had no effect on bone mass (Supporting information Figure S2).

Administration of GCV to 20‐month‐old p16‐3MR mice for 6 months also did not alter bone mass or microarchitecture (Figure 5g,h).

## 3 DISCUSSION

Aging is a critical risk factor for the development of osteoporosis (Almeida et al., 2017; Manolagas, 2018), and cellular senescence has emerged as one of the hallmarks of aging and major contributor to age‐associated diseases, including osteoporosis (Childs et al., 2017; Lopez‐Otin, Blasco, Partridge, Serrano, & Kroemer, 2013). In aged mice, several cell types within bone exhibit markers of senescence (Farr et al., 2016; Kim et al., 2017; Piemontese et al., 2017). The p16‐3MR transgene ablates p16‐expressing cells from a diverse array of cells and tissues including skin, kidney, articular cartilage, hematopoietic system, and arteries (Baar et al., 2017; Chang et al., 2016; Childs et al., 2016; Demaria et al., 2017; Jeon et al., 2017). This maneuver rejuvenates hematopoietic stem cells and counters the development of kidney failure, osteoarthritis, atherosclerosis, and cancer relapse. On the other hand, the elimination of p16‐expressing cells in the skin delays wound healing (Demaria et al., 2014). In the present report, we show that activation of the p16‐3MR transgene efficiently eliminates p16‐expressing cells in the brain and liver of old mice. Unexpectedly, however, activation of the p16‐3MR transgene did not ablate senescent osteocytes in cortical bone. In contrast to our findings, Farr et al. have shown that the INK‐ATTAC transgene effectively decreases the number of senescent osteocytes in old mice (Farr et al., 2017). In that earlier work, the changes in Cdkn2 mRNA levels in cortical bone were very similar to the changes seen by enumeration of osteocytes exhibiting senescence‐associated distension of satellites (SADS). Be that as it may, it remains unknown whether elimination of senescent osteocytes, in and of itself, is responsible for the increase in bone mass seen in the INK‐ATTAC model. The reason(s) for the lack of efficacy of the p16‐3MR transgene in osteocytes remains unclear. Nevertheless, our findings demonstrate that elimination of p16‐expressing cells by the p16‐3MR transgene is tissue selective. Notably, the INK‐ATTAC mediated clearance of senescent cells is also partial and tissue selective (Baker et al., 2016).

In the present work, we focused on macrophages because these cells include the osteoclast progenitors and an increase in osteoclast number is associated with the thinning of cortical bone and the development of cortical porosity in old mice (Piemontese et al., 2017; Ucer et al., 2017). We found that the levels of p16 in macrophage cultures from the bone marrow of old mice were significantly higher as compared to cells from young mice. This finding is in line with evidence that CD14^+^ myeloid‐enriched cell populations isolated from the bone marrow of old mice exhibit elevated levels of p16 and SASP (Farr et al., 2016). F4/80‐positive macrophages in visceral adipose tissue and spleen of old mice also exhibit increased levels of p16 and β‐galactosidase activity (Hall et al., 2016). The activation of the p16‐3MR transgene by GCV abrogated the age‐associated increase in p16 and osteoclastogenic potential of bone marrow‐derived myeloid cells. Albeit, the assays used for this work cannot distinguish whether the changes in cell number are due to altered progenitor number or differentiation capacity. Be that as it may, our findings indicate that cell senescence is a major contributor to the age‐associated increase in osteoclastogenic potential of myeloid cells. Nonetheless, the elimination of the senescent osteoclast progenitors, in and of itself, does not alter endocortical osteoclast number and the loss of bone mass with age.

Cells of the mesenchymal lineage are a critical source of pro‐osteoclastogenic cytokines. We and others have shown earlier that stromal cell cultures obtained from the bone marrow of old mice provide greater support for osteoclastogenesis than cells from young mice (Cao et al., 2005; Kim et al., 2017). Elimination of senescent cells in cultures from old mice, using the senolytic ABT263, decreases the SASP and the pro‐osteoclastogenic effect (Kim et al., 2017). These lines of evidence, along with our present findings, support the notion that senescent cells of the mesenchymal lineage are major contributors to the increase in osteoclast number in the aged skeleton.

We have also shown earlier that a decrease in bone formation contributes to skeletal involution and is associated with a decline in the number of osteoprogenitor and their osteoblast descendants (Almeida et al., 2007; Kim et al., 2017). Specifically, we found that osteoblast progenitor cells, freshly isolated from the bone marrow of old mice, exhibit several markers of senescence (Kim et al., 2017). Nevertheless, osteoblast progenitor senescence is associated with increased p21, but not p16 levels. In view of this earlier finding, we had predicted that activation of the p16‐3MR would not eliminate senescent osteoblast progenitors. In line with our prediction, activation of the p16–3MR transgene by GCV did not alter the markers of senescence or the osteoblastogenic potential of bone marrow‐derived stromal cells. A decrease in osteoblast number is the seminal cellular mechanism for the age‐related loss of trabecular bone in the spine, as indicated by the fact that the number of osteoclasts does not increase with age in this compartment (Almeida et al., 2007). In agreement with the contention that osteoprogenitor senescence is independent of p16, elimination of p16‐expressing cells in old INK‐ATTAC mice does not affect osteoblast number in the trabecular bone of the spine (Farr et al., 2017).

Bone marrow adipocytes increase with age in bone, but the mechanism(s) responsible for this effect and its contribution to age‐related bone loss remains unclear. It is generally accepted that marrow adipocytes arise from mesenchymal progenitor cells (Horowitz et al., 2017). We previously suggested that an increase in oxidized lipids contributes to the age‐related marrow fat accumulation (Almeida, Ambrogini, Han, Manolagas, & Jilka, 2009). In the present study, we found that ablation of p16‐expressing cells in aged p16‐3MR mice decreased the adipogenic potential of bone marrow cells. Similarly, ablation of p16‐expressing cells in aged INK‐ATTAC mice reduces the number of bone marrow adipocytes (Farr et al., 2017). In line with these findings, hepatocyte‐specific senescence leads to fat accumulation in the liver (Ogrodnik et al., 2017). Taken together with the evidence that senescent cells of the mesenchymal lineage were not eliminated by the p16‐3MR transgene, these observations suggest that senescence of other cell types, perhaps within the hematopoietic lineage, contribute via SASP to the increase in adipocyte precursors.

In closing, even though the identity of the senescent cells responsible for the age‐related loss of bone mass remains unclear, the results of the present work strongly suggest that senescent hematopoietic lineage cells are not major culprits. Earlier observations by Farr et al., using the INK‐ATTAC model, indicate that p16‐expressing cells in old mice increase osteoclast number (Farr et al., 2017). Together with our results with the p16‐3MR model, the findings of Farr and colleagues suggest that pro‐osteoclastogenic cytokines of the SASP originating from cells of the osteoblastic lineage contribute to the increase in osteoclast number in the aging skeleton. Because the long‐lived osteocytes are the primary cellular source of the RANKL required for adult bone remodeling (Xiong et al., 2011), accumulation of senescent osteocytes is, likely, a critical mechanism of skeletal aging.

## 4 EXPERIMENTAL PROCEDURES

### 4.1 Mice

p16‐3MR transgenic mice were bred at the University of Arkansas for Medical Science’ (UAMS) Association for the Assessment and Accreditation of Laboratory Animal Care International (AAALAC)‐certified animal facility as described previously (Chang et al., 2016). These mice carry a 3MR (trimodality reporter) fusion protein under the control of the *p16* (also referred to *Cdkn2a*, cyclin‐dependent kinase inhibitor 2a) promoter (Supporting information Figure S1a) (Demaria et al., 2014). The 3MR transgene encodes a fusion protein consisting of Renilla luciferase (LUC), monomeric red fluorescent protein (mRFP), and truncated herpes simplex virus 1 (HSV‐1) thymidine kinase (HSV‐TK), which converts ganciclovir (GCV) into a toxic DNA chain terminator to selectively kill HSV‐TK‐expressing senescent cells (Supporting information Figure S1a). Middle‐aged C57BL/6J mice were purchased from Jackson Laboratory (Bar Harbor, MA, USA). Mice were randomly assigned to 3 to 5 mice per cage, received food and water *ad libtum,* and were housed at the UAMS AAALAC‐certified animal facility. Mice with tumors and/or leukemia were excluded from experiments and analyses. All animal work was approved and done in accordance with the UAMS Animal Care and Use Committee.

### 4.2 Micro‐CT

Bones were cleaned of adherent tissue, fixed in Millonig's (Leica Microsystems), and stored in 100% ethanol, loaded into 10‐mm diameter scanning tubes, and imaged (micro‐CT40; Scanco Medical, Brüttiselen, Switzerland), and the vertebral and femoral cancellous bone was analyzed as described previously (Martin‐Millan et al., 2010). Scans were performed at medium resolution (12 µm isotropic voxel size) for quantitative determinations and integrated into 3‐D voxel images (1,024 × 1,024 pixel matrices for each individual planar stack). A Gaussian filter (sigma = 0.8, support = 1) was applied to all analyzed scans. Key parameters were X‐ray tube potential = 55 kVp, X‐ray intensity = 145 µA, integration time = 200 ms, and threshold =200 mg/cm^3^. Cortical dimensions were determined using 18 and 23 slices at the femoral mid‐diaphysis. Total and medullary area and circumference measurements were calculated from these slices. For cortical porosity measurements, slices were analyzed from a point immediately distal to the third trochanter to the primary spongiosa. After defining endosteal and periosteal boundaries, an additional image processing script (“peel‐iter = 2”) was used to eliminate false voids caused by imperfect wrap of the contours to the bone surface. Images were binarized with a threshold of 365 mg/cm^3^, and overall porosity determined with the “cl_image” script to obtain bone volume and void volume. To avoid inclusion of osteocyte lacunae and canalicular space, void volumes <31,104 µm^3^ (18 voxels) were excluded in the determination of porosity.

### 4.3 Biomechanical testing

The load‐bearing properties of the first lumbar vertebrae (L1) were measured using a single‐column material testing machine and a calibrated tension/compression load cell (model 5542; Instron Corp, Canton, MA, USA) as previously described (Almeida et al., 2007).

### 4.4 Bone histology and histomorphometry

Freshly dissected bones were fixed in Millonig's overnight, transferred to 100% ethanol, and embedded undecalcified in methyl methacrylate. Histomorphometric examination was performed in longitudinal sections using the OsteoMeasure Analysis System (OsteoMetrics, Inc., Decatur, GA, USA) as previously described (Piemontese et al., 2017). The terminology used in this study has been recommended by the Histomorphometry Nomenclature Committee of the American Society for Bone and Mineral Research.

### 4.5 Osteoblast differentiation

Total bone marrow cells pooled from 4–7 mice from each group were cultured with 20% FBS, 1% PSG, and 50 µg/ml of ascorbic acid (Sigma) in 10‐cm culture dishes for 5 days. Half of the medium was replaced every 3 days. Cells were then cultured with 10% FBS, 1% PSG, 50 µg/ml of ascorbic acid, and 10 mM β‐glycerophosphate (Sigma) for 21 days. Mineralized matrix was stained with 40 mM alizarin red solution (Sigma).

### 4.6 Osteoclast differentiation

BMMs were obtained as described previously (Bartell et al., 2014). Whole bone marrow cells were cultured with 10% FBS, 1% PSG after removing red blood cells by ACK buffer (0.01 mM EDTA, 0.011 M KHCO3, and 0.155 M NH4Cl, pH 7.3) for 24 hr in the presence of 10 ng/ml of M‐CSF (R&D Systems). Nonadherent cells were cultured in Petri dishes with 30 ng/ml of M‐CSF for 3–4 days to generate BMMs. To generate osteoclasts, BMMs were cultured with 30 ng/ml of M‐CSF and 30 ng/ml of RANKL (R&D Systems) for 4–5 days. Cells were fixed with 10% neutral buffered formalin for 10 min and stained for tartrate‐resistant acid phosphatase (TRAP), using the Leukocyte Acid Phosphatase Assay Kit, following the manufacturer's instructions (Sigma‐Aldrich). An osteoclast was defined as a multinuclear TRAP‐positive cell. For all assays, cells were plated in triplicate.

### 4.7 Adipocyte differentiation

Bone marrow‐derived stromal cells were cultured to 70% confluence, and the media supplemented with rosiglitazone (5 nM/ml) or with 3.3% BSA in PBS as vehicle control. Medium was changed every 3 days. After 11 days, cells were fixed in 10% formalin in PBS, rinsed, and stained for 30 min with 0.15% Oil Red O (Sigma) in a 55:45 mix of isopropanol and water. Cells were counterstained with 0.5% methyl green (Fisher Scientific) in 0.1 M sodium acetate, pH 4. Oil Red O staining was quantified after extraction of the dye with 1 ml isopropanol and absorbance determination at 490 nm.

### 4.8 Western blot analysis

Osteocyte‐enriched femoral cortical bone was obtained as described previously (Piemontese et al., 2017). The tissue fragments including femoral bone, brain, and liver were immediately frozen in liquid nitrogen and pulverized. Proteins were extracted with a buffer containing 20 mM Tris‐HCL, 150 mM NaCl, 1% Triton X‐100, protease inhibitor mixture, and phosphatase inhibitor cocktail (Sigma‐Aldrich) on ice for 30 min and then keep on −80°C for overnight. The cultured cells were washed twice with ice‐cold PBS and lysed with the same buffer as described above. Protein concentration of bone extract and cultured cells was determined using the DC Protein Assay Kit (Bio‐Rad). The extracted protein (20–40 µg per sample) was subjected to 10 or 15% SDS‐PAGE gels and transferred electrophoretically onto PVDF membranes. The membranes were blocked in 5% fat‐free milk/Tris‐buffered saline for 120 min and incubated with each primary antibody followed by secondary antibodies conjugated with horseradish peroxidase. Mouse monoclonal antibodies against p21 (Santa Cruz Biotechnology, sc‐6246, 1:500), β‐actin (Santa Cruz Biotechnology, sc‐81178, 1:2000), γ‐H2AX (Millipore #05‐636, 1:5000), p16 (Santa Cruz Biotechnology, sc‐1661, 1:2000), and goat polyclonal antibody for GATA4 (Santa Cruz Biotechnology, sc‐1237, 1:500) were used to detect their corresponding protein levels. The membranes were subjected to Western blot analysis with ECL reagents (Millipore). The quantification of the intensity of the bands in the autoradiograms was performed using a VersaDocTM imaging system (Bio‐Rad).

### 4.9 Quantitative RT‐PCR (qRT‐PCR)

Total RNA from cell cultures was extracted with TRIzol reagent (Invitrogen) and reverse‐transcribed using the High‐Capacity cDNA Archive Kit (Applied Biosystems) according to the manufacturer's instructions. TaqMan quantitative real‐time PCR was performed using the following primers from Applied Biosystems: Cdkn2a (Mm00494449_m1); Il‐6 (Mm00446190_m1); TNF‐a (Mm00443258_m1); Rankl (Mm00441908_m1); Mmp‐13 (Mm00439491_m1); Il‐1α (Mm99999060_m1); p21 (Mm00432448_m1). All reactions were run in triplicate and target gene expression was calculated by normalizing to the housekeeping gene ribosomal protein S2 (Mm00475528_m1) using the ∆Ct method (Livak & Schmittgen, 2001).

### 4.10 Statistical analysis

The data displayed normal variance. The experiments were not randomized, except for the animal experiments as described above. The experimental sample size (n) of each group is described in each corresponding figure legend. The data were analyzed by analysis of variance (ANOVA) or Student's *t* test (independent samples, two‐sided) using GraphPad Prism 7 from GraphPad Software, after determining that the data were normally distributed and exhibited equivalent variances. All experiments were repeated at least twice. Statistical significance was set at a *p* < 0.05. Error bars in all figures represent *SD*.

## CONFLICT OF INTEREST

J. Campisi and D.Z. are co‐founders of and advisors to Unity Biotechnology, which develops small‐molecule senolytic drugs for age‐related disease.

## AUTHOR CONTRIBUTIONS

D.Z., H.N.K., and M.A. designed the experiments and analyzed the data. J.C. performed animal work. H.N.K., and L.H. performed in vitro studies. S.I. performed bone histology. J. Campisi provided reagents and technical advice. H.N.K., S.C.M., D.Z., and M.A. discussed results. H.N.K. and M.A. wrote the paper. All the authors revised the manuscript.
