# Supplementary figures and images for: Elimination of senescent osteoclast progenitors has no effect on the age‐associated loss of bone mass in mice
Source: Aging Cell. 2019 Feb 17;18(3):e12923. doi: 10.1111/acel.12923 (PMC6516158; doi:10.1111/acel.12923)

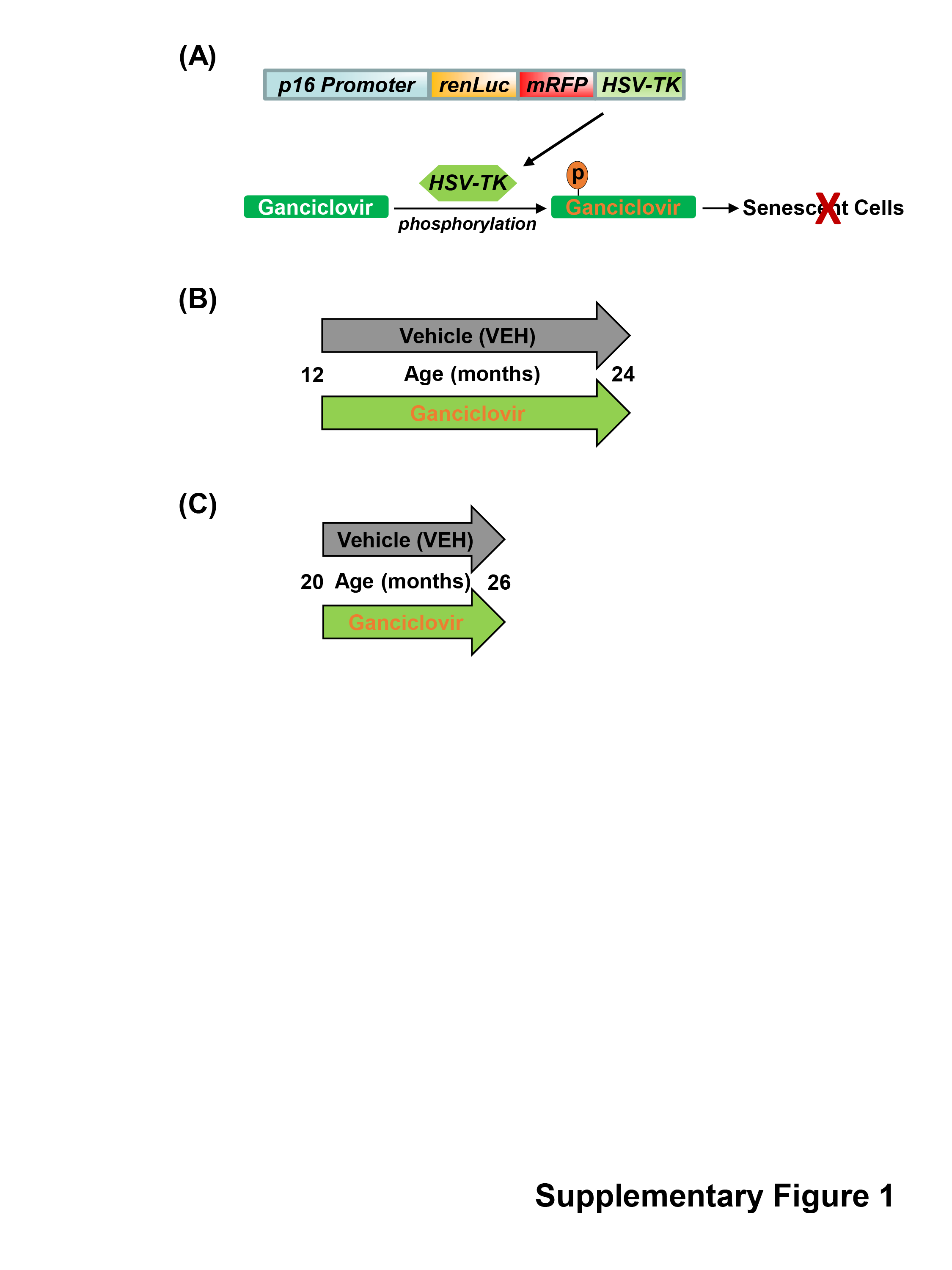

Supplement: Supplementary file 1 [file ACEL-18-e12923-s001.tif]

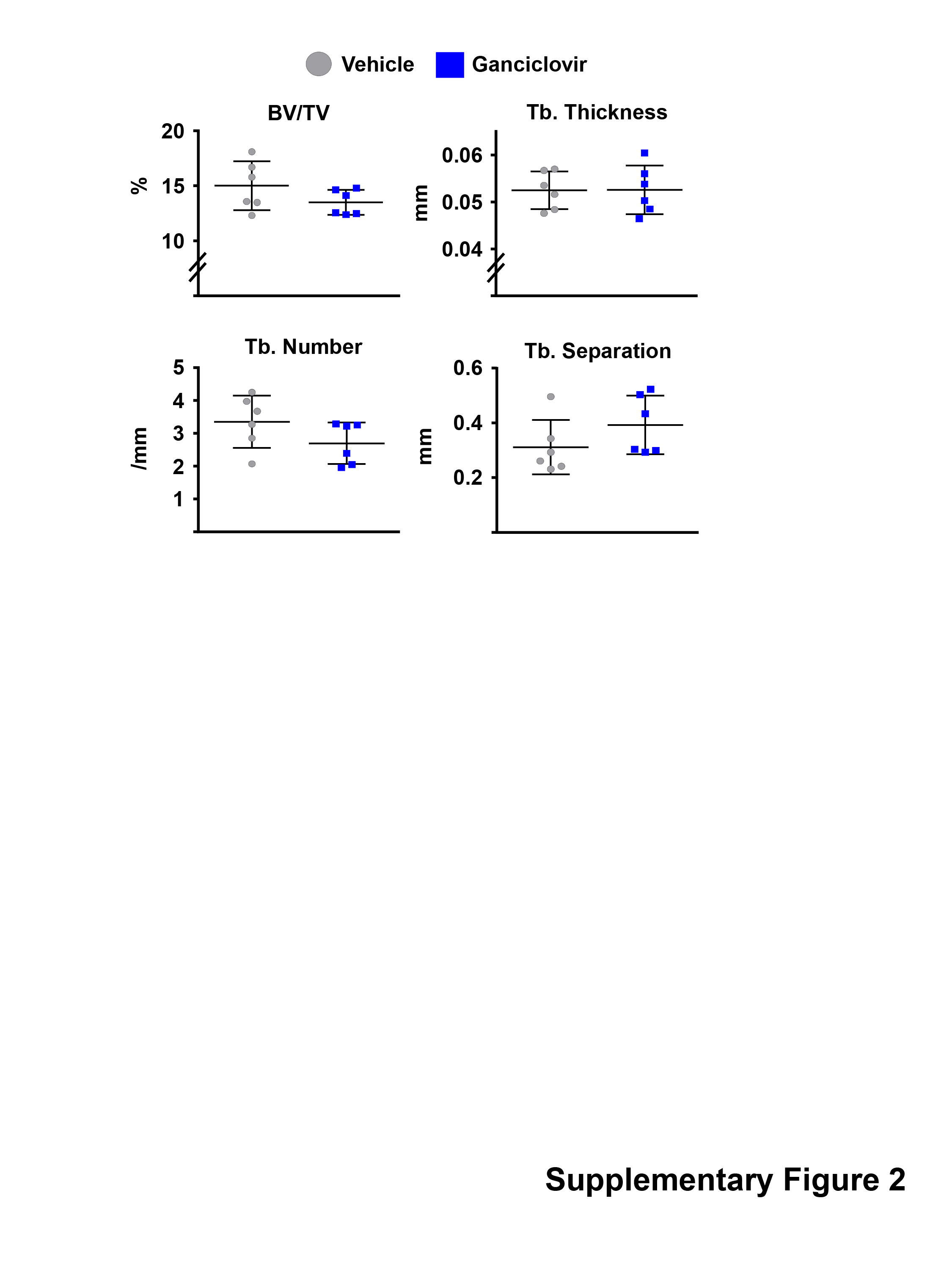

Supplement: Supplementary file 2 [file ACEL-18-e12923-s002.tif]

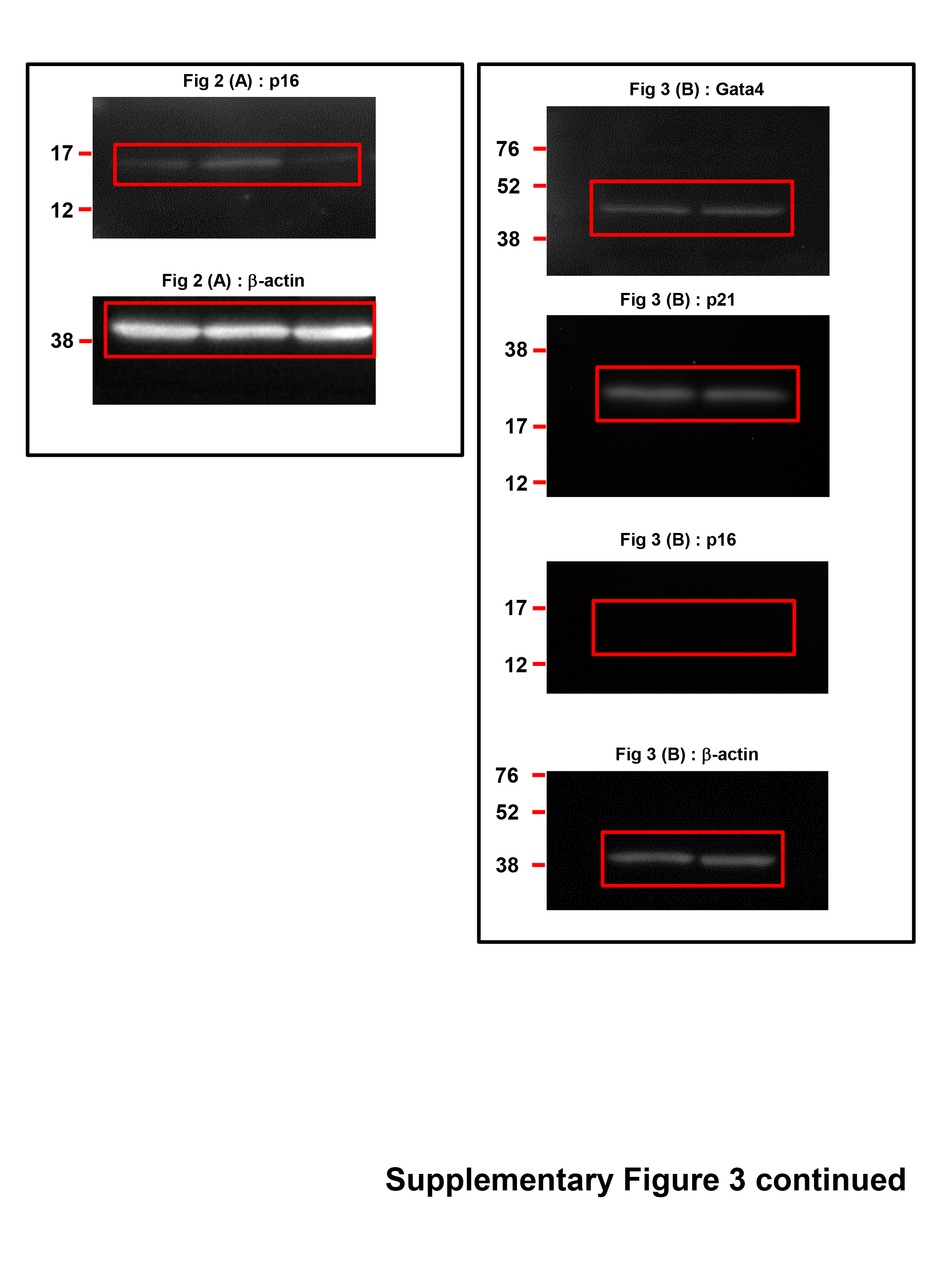

Supplement: Supplementary file 3 [file ACEL-18-e12923-s003.tif]

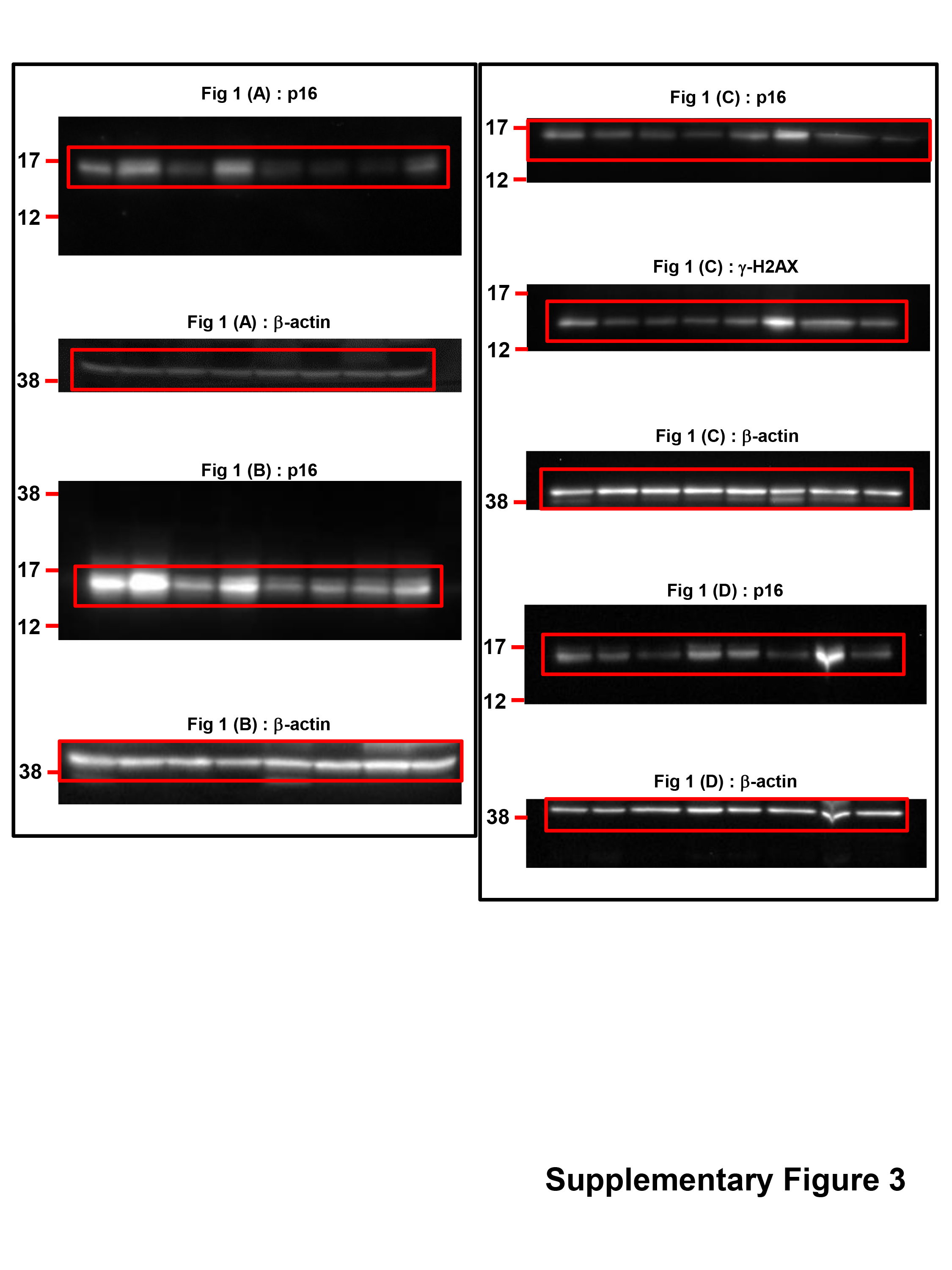

Supplement: Supplementary file 4 [file ACEL-18-e12923-s004.tif]
